# Hand Motions Reveal Attentional Status and Subliminal Semantic Processing: A Mouse-Tracking Technique

**DOI:** 10.3390/brainsci13091267

**Published:** 2023-08-31

**Authors:** Kunchen Xiao, Anqi Zhang, Jingke Qu, Feifei Deng, Chenyan Guo, Takashi Yamauchi

**Affiliations:** 1 Institute of Brain and Psychological Sciences, Sichuan Normal University, Chengdu 610066, China; 2 Department of Psychological and Brain Sciences, Texas A&M University, College Station, TX 77843-4235, USA

**Keywords:** mouse-tracking, cursor motion, attention, congruency effects, subliminal semantic processing, area under the curve

## Abstract

Theories of embodied cognition suggest that hand motions and cognition are closely interconnected. An emerging technique of tracking how participants move a computer mouse (i.e., the mouse-tracking technique) has shown advantages over the traditional response time measurement to detect implicit cognitive conflicts. Previous research suggests that attention is essential for subliminal processing to take place at a semantic level. However, this assumption is challenged by evidence showing the presence of subliminal semantic processing in the near-absence of attention. The inconsistency of evidence could stem from the insufficient sensitivity in the response time measurement. Therefore, we examined the role of attention in subliminal semantic processing by analyzing participants’ hand motions using the mouse-tracking technique. The results suggest that subliminal semantic processing is not only enhanced by attention but also occurs when attention is disrupted, challenging the necessity of facilitated top-down attention for subliminal semantic processing, as claimed by a number of studies. In addition, by manipulating the color of attentional cues, our experiment shows that the cue color per se could influence participants’ response patterns. Overall, the current study suggests that attentional status and subliminal semantic processing can be reliably revealed by temporal–spatial features extracted from cursor motion trajectories.

## 1. Introduction

### 1.1. Background

Regarding the relationship between consciousness and attention, a classic theory is that consciousness and unconsciousness have distinct features: conscious processing is elaborate, flexible, and controlled by attention, while unconscious processing is superficial, stereotypical, and independent of attention [1,2]. On the contrary, recent Global Workspace Theory argues that unconscious processing is more elaborate and flexible than previously regarded [3]. Research on masked priming suggests that semantic processing, which is conventionally considered relatively elaborate, can take place at a subliminal level. And subliminal semantic processing is modulated by top-down attention [4]. However, the role of attention in unconsciousness is controversial because of inconsistent results; the unsatisfactory reproducibility could be attributed to the lack of sensitivity in the traditional reaction time measure to reveal subtle unconscious cognition [5]. Thus, the current study proposes a novel methodology, which tracks participants’ hand motions in choice-reaching tasks, to investigate the influence of attention on subliminal semantic processing.

According to theories of embodied cognition, cognition and body motion are closely interconnected [6], such as the interplay of visual perception and motion processing [7]. In particular, choice-reaching by hand (i.e., moving hands to select an intended target) is a fluid process of decision making, where muscles and neurons coordinate dynamically in a continuous feedback loop: higher cortical systems make a coarse motor plan, and local sensorimotor subsystems adjust the hand motion by adapting to sensory feedback in real-time [8,9]. If these sensorimotor coordination processes are intervened by cognitive conflicts, the motor plan for choice-reaching will be restructured (e.g., speed change or making a turn), modifying temporal–spatial features of the hand motion path [10,11,12]. Thus, hidden cognitive states, such as implicit semantic processing, can be revealed by how people move their hands during choice-reaching [13,14].

An emerging technique has been developed to track users’ hand motion by recording the cursor movements of a computer mouse [15,16]. Temporal–spatial features of the cursor motion paths reflect participants’ dynamic decision-making processes [17,18]. The mouse-tracking technique has been widely used in research on semantic processing [19], social judgment [20], and affective computing [21,22]. Recent research further integrates mouse-tracking into neuropsychological tests and finds that mouse-tracking benefits psychopathological assessment of stress [23,24], impulsivity [25], and attention deficit hyperactivity disorder [26,27,28]. Particularly, the cursor motion features are powerful in uncovering hidden cognitive conflicts [29,30,31], such as the implicit social stereotype [32], attitudinal ambivalence [33], uncertainty in economic choices [34], and syntactic incongruency [35]. Thus, the mouse-tracking technique provides a promising methodology to shed light on unconscious cognition.

### 1.2. Subliminal Semantic Processing and Attention

**Figure 1:**
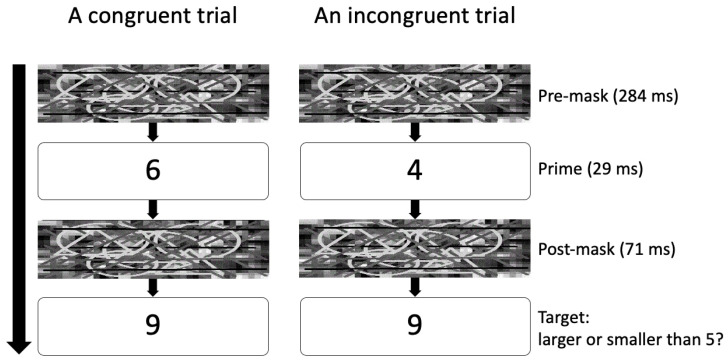
An illustration of the “congruency effect”. The participants’ task was to judge whether the target number was larger or smaller than 5. Before presenting the target, a priming digit was displayed briefly for 29 ms, sandwiched by masks to make the priming digit subliminal. The response time was shorter for congruent trials than incongruent trials, which is the “congruency effect”.

A typical unconscious cognitive conflict is semantic incongruency. For example [36], participants judged if a target digit was larger or smaller than five, preceded by a subliminal priming digit (i.e., the “prime”), which was masked (Figure 1). Although the prime was invisible, it influenced participants’ semantic judgment: the response time was shorter for “congruent” trials (i.e., priming and target digits were both smaller or both larger than five) than “incongruent” trials (e.g., prime = 4, target = 9), known as the “congruency effect”.

The congruency effect demonstrates that semantic information of the subliminal stimuli can been processed without participants’ awareness [37,38]. Furthermore, participants’ attentional status was manipulated by occasionally presenting a cue: in the “cued” condition, an attentional cue (i.e., a green square) forecasting the impending target facilitated top-down attention, while in the “uncued” condition, the pre-target duration varied randomly with no cue presented to disrupt participants’ temporal attention window [39]. The congruency effect was observed only in the cued condition, suggesting that top-down attention enhanced relevant types of subliminal semantic processing [40,41].

However, the exact role of attention in unconscious semantic processing is controversial [42,43]. Some research argued that assistance from attention was necessary for semantic processing to occur at a subliminal level [44,45,46]. Meanwhile, other studies only confirmed that attention modulated semantic priming but found no solid evidence for the necessity of attention [47,48]. On the other hand, some studies failed to replicate the modulation effect of attention on unconscious processing [49,50,51]. And subliminal congruency effects were also observed in the near-absences of attention [52,53]. For example, the gender of masked faces could be reliably identified with little attention, whereas non-face stimuli could not be unconsciously distinguished [54,55,56]. And masked pictures of natural scenes could be correctly categorized when the focal attention was suppressed [57,58].

The inconsistency of evidence regarding the role of attention in subliminal semantic processing may arise from the way that congruency effects have been evaluated. The traditional method of measuring response time captures only two data points for each trial: the onset and end times. This approach overlooks any cognitive process that takes place in between. Relying solely on the temporal measurement provides limited information about the intricate cognitive processes that unfold rapidly in real-time, which are yet fundamental for subliminal semantic processing to occur [59]. To probe these dynamic processes, a measurement recording fine-tuned data points corresponding to the continuous cognition-sensorimotor coordination is needed. In this case, the mouse-tracking data are beneficial because they integrate temporal–spatial information that characterizes participants’ dynamic cognitive processes [60,61]. Though temporal data alone may underestimate semantic congruency effects, the temporal–spatial features of cursor motion help tease apart subliminal semantic processing [62,63].

### 1.3. Summary of Previous Research

A number of studies suggest that subliminal semantic congruency effects measured by response time are generally small and difficult to replicate [64,65,66]. One reason for the mediocre reproducibility could be the inadequate sensitivity in the measurement to detect the nuance of unconscious processing [67,68]. Indeed, substantial research corroborates the advantage of mouse-tracking measurement over response time, particularly for uncovering implicit cognitive conflicts [31,59,62]. For example, when participants judged whether a face belonged to a white or black person, their cursor motion trajectories were attracted to the unintended label “white” without awareness if an atypical black face was presented [20]. Similarly, semantic congruency effects can be reliably measured by the attraction of cursor trajectories toward the unintended alternative option, which is quantified by the “area under the curve” (AUC) of the cursor motion curves [59,69]. Research comparing mouse-tracking and response time shows that semantic congruency effects measured by AUC were significantly larger than those by response time [62,70,71]. Thus, the absence of semantic congruency effects in the uncued condition in the Naccache et al. [39] and follow-up studies could stem from the insufficient resolution in the response time measurement to reliably expose subliminal processing.

Another unresolved issue is the role of the attentional cue. It is possible that the cue not only facilitated top-down attention but also worked as another indirect prime. Previous research shows that the color of a visual cue is closely associated with semantic priming [72]; for example, a green-color priming picture facilitates participants’ positive responses while a red-color picture polarizes negative responses [69,71,73]. Because the green/red colors are often associated with go/no-go signals, the cue color per se could influence the pattern of responses. Thus, the exact role of the attentional cue needs further clarification.

### 1.4. Purposes of the Current Study

To address these unresolved issues, the current study employs the mouse-tracking paradigm rather than the response time method to examine the role of attention in subliminal semantic processing. Following the experimental procedures in the Naccache et al. study [39], participants’ attentional status was manipulated by occasionally presenting an attentional cue. To investigate the influence of cue color, the experiment contrasted two between-participant conditions: one group of participants received only a green cue (i.e., the green condition), while the other group received only a red cue (i.e., the red condition). The experiment seeks to clarify (1) whether subliminal semantic processing occurs when attention is disrupted, (2) the influence of cue color on subliminal semantic processing, and (3) whether subliminal semantic processing and attentional status can be revealed by characteristics of cursor motion trajectories.

## 2. Materials and Methods

### 2.1. Overview of the Experiment

We combined the procedure of the second experiment in the Naccache et al. (2002) research and the mouse-tracking technique [39]. Participants indicated if a target digit was larger or smaller than 5, and a masked priming digit was presented prior to the target. In some trials, an attentional cue (a green square) was shown to signal impending digits. Unlike the original study, however, we added a between-subject condition to manipulate the cue color—in addition to the green square cue, participants received a red square cue in another condition. In this manner, we investigated the role of the cue in a 2 × 2 × 2 factorial design, in which congruency (congruent vs. incongruent—within-subject factor), cue (cued vs. uncued—within-subject factor), and cue color (green vs. red—between-subject factor) were contrasted. If top-down attention enhances subliminal processing of the digits, both red and green cues should elicit more substantial priming effects relative to uncued conditions. If the cue color influences semantic priming, red and green cues should lead to different patterns of congruency effects.

### 2.2. Participants

Eighty undergraduate students participated in the experiment for course credit. They were randomly assigned to the green (*n* = 40) or red (*n* = 40) condition. All participants’ response accuracy was above 80%. Three participants failed to finish the experiment, and data from 77 participants (*n* = 37 in the green condition; *n* = 40 in the red condition) were analyzed. There were 18 males and 19 females in the green condition while 18 males and 22 females were in the red condition. Their ages ranged from 17 to 23 (*M*_green_ = 19.68, *SD*_green_ = 2.12; *M*_red_ = 20.08, *SD*_red_ = 1.95). A meta-analysis shows that 23 studies on masked semantic priming (within-subject design) had a mean sample size of twenty and a mean effect size of 0.8 with a 95% confidence interval [0.60, 1.00] [5]. Using this pooled estimate of effect size, our prospective power analysis indicates that a sample size above 37 for each condition is sufficient to detect a congruency effect size of 0.6 at an alpha level of 0.05 with a power of 0.90.

### 2.3. Materials and Apparatus

We employed single-digit numbers (i.e., 1, 4, 6, or 9) as the prime and target. The display refresh rate of the monitor was 70 Hz, and the resolution was 1280 × 720. The computer program was developed using Microsoft Visual Studio v.15.0, and the computer mouse was a Logitech M100 Corded Mouse.

### 2.4. Experimental Procedure

**Figure 2:**
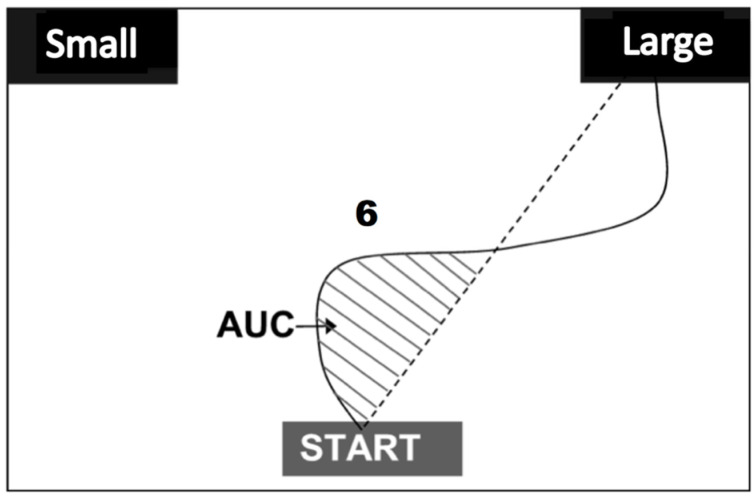
An illustration of the area under the curve (AUC). In each trial, the AUC (shaded area) is measured as the number of pixels enclosed by the dashed direct line connecting the starting and ending points and the actual cursor curve that goes over the direct path toward the unselected option. Any area that goes over the direct path toward the selected choice is subtracted as negative AUC. The cursor always starts from the center of the “START” button and ends where participants click one of the two options.

In the mouse-tracking paradigm, participants move a computer mouse to select a choice, and the area under the curve (AUC) is measured in each trial (Figure 2). A smaller AUC denotes a more straightforward and certain response, while a larger AUC reflects uncertainty during the response and more distraction from the unselected option [15,30,59]. To depict the curve, the computer program obtains the location of the cursor on the monitor screen every 15 ms; all data points in each trial are standardized into 101 steps with a linear interpolation method.

The experiment had two phases: a number judgment task and an awareness test. In the number judgment task, participants were assigned randomly to one of the two conditions: the green condition (i.e., the cue color is green) or the red condition. The procedure followed Experiment 2 in the Naccache et al. study [39], except that the red condition was added. 

**Figure 3:**
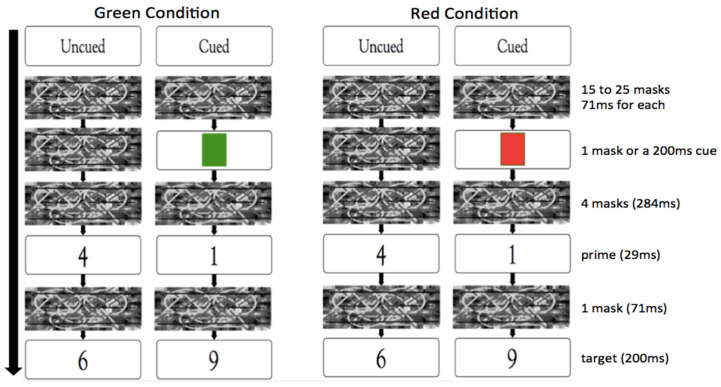
The procedure in the green and red conditions. The two conditions differed only in the cue color.

In each trial, a mask (i.e., a black-white rectangle doodled with circles and lines) was displayed for a random number of frames (i.e., 15 to 25 frames) in the center of the screen; each frame lasted for a fixed duration of 71 ms. Thus, this pre-mask lasted randomly from 1065 ms to 1775 ms with a 71 ms increment. Succeeding the pre-mask, a cue (i.e., a 200 ms green/red square) in the cued trials or one frame of the mask (71 ms) in the uncued trials was presented. The cue was green for participants assigned to the green condition and red for those to the red condition. Following the cue (or the 71 ms mask), 4 frames of 71 ms masks were displayed, succeeded by a 29 ms priming digit (i.e., 1, 4, 6, or 9), and then a 71 ms post-mask was presented. Following that, a target number (i.e., 1, 4, 6, or 9) was presented for 200 ms (Figure 3). Participants were required to judge if the target number was smaller or larger than five and move a computer mouse to select the “Small” or “Large” button on the top left/right locations on the screen (Figure 2). The locations of the “Small” and “Large” buttons were counterbalanced among participants.

In this number judgment task, there were 96 cued trials and 192 uncued trials, presented in random order. Half of the trials were congruent (i.e., the target and prime were both smaller or larger than five; e.g., prime = 4, target = 1), and the remaining half were incongruent (e.g., prime = 4, target = 6). Before the number judgment task, there was a practice session, where 96 trials (32 cued and 64 uncued trials) were performed. Trials with a response time longer than 5000 ms were excluded from the data analysis (0.6% of total trials).

Following the number judgment task, an awareness test was given, where trials (192 trials in total) were the same as in the number judgment task. Participants were explicitly informed that a priming digit would flash briefly and were required to judge if the prime was visible and whether it was larger or smaller than 5, instead of judging the target digit [37]. The *d*’ was calculated for each participant to check the visibility of priming digits: correctly indicating a prime as larger than five was defined as “hit” and choosing “Large” when the prime was smaller than five as “false alarm”. We applied linear regression analyses for congruency effects on *d*’s to examine the extent to which the visibility of primes would contribute to priming effects [37].

## 3. Results

### 3.1. The Three-Way ANOVA

The experiment was a 2 (cue: cued, uncued; within-subject) × 2 (cue color: green, red; between-subject) × 2 (congruency: congruent, incongruent; within-subject) factorial design. The dependent variable was the area under the curve (AUC).

A three-way ANOVA (cue color × cue × congruency) revealed significant impacts of cue color (*F*(1, 75) = 5.62, *MSE* = 48,246,665.40, *p* = 0.02, partial *η*^2^ = 0.07), congruency (*F*(1, 75) = 53.43, *MSE* = 953,709.97, *p* < 0.001, partial *η*^2^ = 0.42), and interaction between congruency and cue (*F*(1, 75) = 13.54, *MSE* = 618,393.72, *p* < 0.001, partial *η*^2^ = 0.15). The three-way interaction was not significant (*F* < 1).

The congruency effects were prominent, suggesting that the masked priming numbers can be processed at a semantic level without much awareness. Below, we report the impacts of presenting an attentional cue and those of the cue color on congruency effects, respectively.

### 3.2. The Impact of Presenting an Attentional Cue

**Figure 4:**
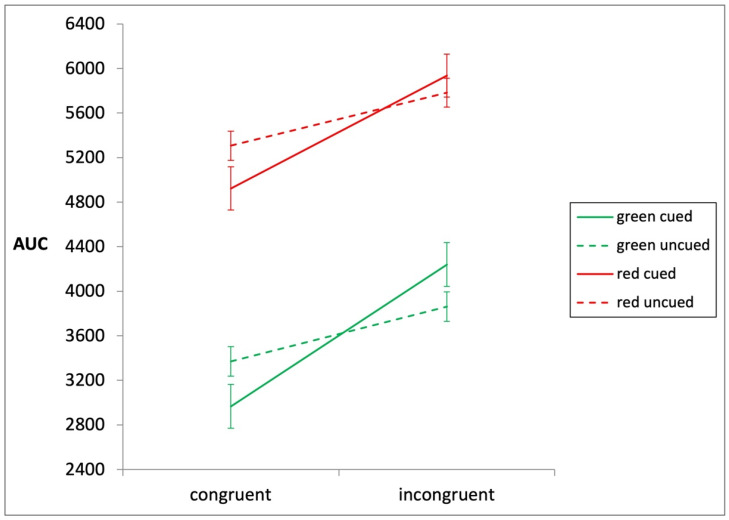
The average AUCs of congruent and incongruent trials are shown for cued and uncued conditions in the green and red conditions, respectively. The AUCs were measured by amounts of pixels on the screen.

The significant interaction between congruency and cue (*F*(1, 75) = 13.54, *MSE* = 618,393.72, *p* < 0.001, partial *η*^2^ = 0.15) shows that the congruency effects were larger in the cued than the uncued conditions (Figure 4), which means presenting an attentional cue enhanced the congruency effects. It is noticeable that congruency effects were still robust in uncued conditions (Figure 4), suggesting that the masked numbers elicited semantic processing even when the temporal attentional window was disrupted (i.e., in the uncued condition).

### 3.3. The Impact of Cue Color

The cue color influenced overall AUCs. The average AUC in the red condition (*M* = 3611.62, *SD* = 3383.87) was larger than that in the green condition (*M* = 5506.54, *SD* = 3724.76); (*F*(1, 75) = 5.62, *MSE* = 4,8246,665.40, *p* = 0.02, partial *η*^2^ = 0.07). Meanwhile, the interaction between cue and congruency was significant in the green condition (*F*(1, 36) = 11.00, *MSE* = 515,318.83, *p* = 0.002, partial *η*^2^ = 0.23) but not in the red condition (*F*(1, 39) = 4.04, *MSE* = 713,539.77, *p* = 0.051, partial *η*^2^ = 0.09).

### 3.4. Characteristic of Average Motion Trajectories: Congruent vs. Incongruent Trials in Green and Red Conditions

**Figure 5:**
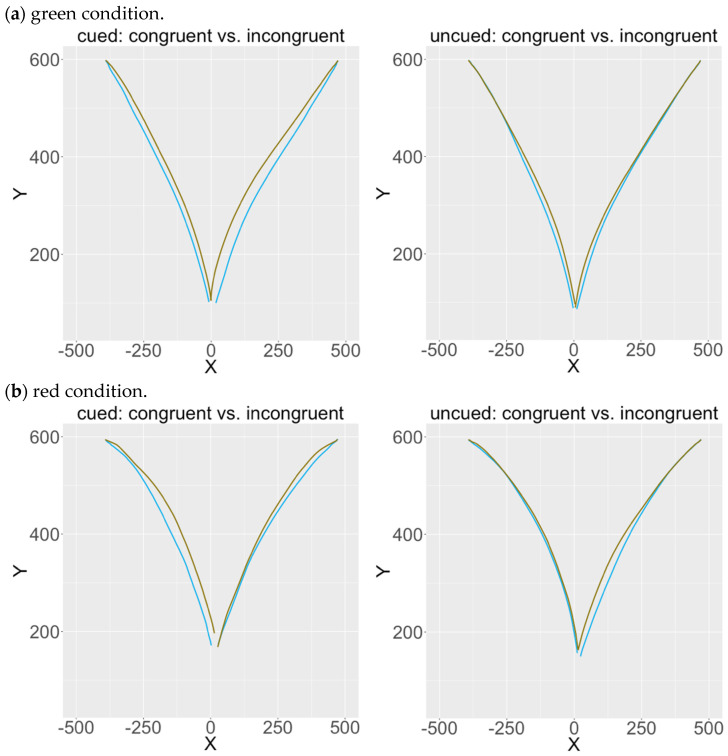
Average trajectories in the green condition (**a**) and the red condition (**b**), with the cued condition on the left panel and the uncued condition on the right panel. Trajectories in light blue represent congruent trials while those in light brown represent incongruent trials. All trajectories start from the lower-middle location and travel to one of the two options either on the upper-left corner (e.g., “Small”) or on the upper-right corner (e.g., “Large”) of the screen, depending on participants’ choices. The congruency effect is illustrated by the distance between the congruent and the incongruent trajectories toward the same ending location, which corresponds to the difference in average AUCs between congruent and incongruent trials. The X and Y axes denote amounts of pixels on the screen.

Figure 5 below shows the average cursor motion trajectories in the cued/uncued conditions as well as in the green (a) and red (b) conditions. Blue trajectories are for congruent trials while brown trajectories are for incongruent trials. The congruency effect is illustrated by the distance between the congruent and the incongruent trajectories toward the same ending location, which corresponds to the difference in average AUCs between congruent and incongruent trials (i.e., the size of congruency effects). The congruency effect is larger in the cued than the uncued conditions yet still present in the uncued condition (Figure 5), suggesting that subliminal semantic processing occurs in the near-absence of top-down attention.

### 3.5. Awareness Analyses

In the awareness test, no participant could correctly report any priming digit. To further examine the visibility of primes, we calculated the *d*’s in the awareness test [37]. The *d*’s were statistically indistinguishable from zero: in the green condition, *M* = 0.001, *t*(36) = 0.10, and *p* = 0.923; in the red condition, *M* = 0.004, *t*(39) = 0.49, and *p* = 0.625; these results suggest that participants had little awareness of primes. Linear regressions showed that *d*’s could not predict congruency effects; thus, congruency effects were unlikely to be impacted by the visibility of primes (Table 1 and Table 2). The intercepts were higher than zero at null *d*’, indicating that congruency effects were significant at a subliminal level [37]. 

## 4. Discussion

The overall AUCs in the red condition were larger than those in the green condition, suggesting that cue color per se influenced subliminal semantic processing. Given that color is closely associated with semantic priming (e.g., the red color is often associated with negative connotations like “alarm” and “stop”) [72,73], the red cue could make participants more cautious and hesitant to respond, resulting in larger AUCs. The same rationale could explain the smaller AUC in the green condition—positive meanings implied by the green color, such as “safe” and “go”, might have prompted more straightforward responses [69,71]. The results suggest that researchers manipulating attentional status with visual cues shall proceed with caution and minimize the influence of cue color on priming effects.

The relationship between attention and consciousness is a long-debated core issue in human cognition. The classic theory claims that conscious processing is guided by attention while unconscious processing is autonomous, stereotypical, and independent of attention [74,75]. In contrast, recent research suggests that attention can orient to invisible stimuli and influence unconscious processing. Accumulated evidence suggests that top-down attention can modulate subliminal processing at a semantic level [76]. According to the Global Workspace Theory (GWT), unconscious processing needs assistance from top-down attention to reach a semantic level [77,78]. More specifically, subliminal semantic information is automatically coded by specialized neural modules in the peripheral workspace and sent into the global workspace; the top-down control mechanism in the global workspace allocates more cognitive resources to the attended semantic information, leading to enhanced subliminal priming, while unattended subliminal processing quickly fades away [79].

Challenging this view, the current study finds significant congruency effects in the uncued conditions, suggesting that unconscious information is processed at a semantic level even with little support from attention. Thus, unconscious processing need not be sustained by attention to produce semantic priming [62,70]. On the other hand, congruency effects are larger in the cued than in the uncued conditions, consistent with previous research on GWT [39,80]. Therefore, the current study rather proposes a revision than disproves the GWT: in the global workspace, the top-down control mechanism does enhance attended unconscious information; meanwhile, at least some types of semantic processing, though not sustained by attention, persist for a notable duration in a bottom-up manner instead of immediately vanishing.

The current study adds solid evidence to the robustness and flexibility of subliminal semantic processing, contrary to the traditional opinion that unconscious processing is short-lived and stereotypical [81,82]. Given the close interplay of top-down attention and subliminal information, the boundary between conscious control and unconscious processing becomes vague [83]. Since hand movements reflected subliminal processing, the underlying mechanism linking overt body motions and implicit cognition deserves further clarification to enrich the embodied cognition theory.

Overall, the mouse-tracking measure (e.g., AUC) shows advantages over response time to detect implicit cognitive conflicts [71]. Conventionally, decoding real-time cognitive activities relies on brain imaging techniques (e.g., EEG and fMRI), which require costly devices and cumbersome settings. In contrast, the mouse-tracking technique is an affordable, easy-to-use method adaptive to a broad range of user environments [59]. A computer mouse is one of the most ubiquitous devices and the cursor motion is traced by time-stamped x-y coordinates, minimizing costs for data collection and analysis. Given that subthreshold motor control processes cannot be revealed by button responses yet can be detected by BOLD signals [84], it is also possible that similar mechanisms can be measured with mouse-tracking. Thus, the mouse-tracking technique provides a promising tool to dissect dynamic cognitive processes and contribute to brain sciences in future research [16,85].

Admittedly, the current study comes with limitations. First, since disrupted attention in the uncued condition did not guarantee a complete absence of attention, we cannot rule out the possibility that minimal attention still played a role there. Rather, because semantic priming was present in both cued and uncued conditions, assistance from attention is unlikely to be essential for subliminal semantic processing. Second, the main effect of cue color was based on between-group comparisons, which could be confounded with potential between-group individual differences in movement styles, if any. Although the between-group design helps prevent red and green colors from working against each other, future research shall consider within-group design to re-examine the color effect.

## 5. Conclusions

In conclusion, the current study investigates the role of attention in subliminal semantic processing with the mouse-tracking technique and provides three critical messages. First, while attention enhances subliminal semantic processing, the cue color per se influences semantic priming effects. Second, top-down attention is unlikely to be a prerequisite for subliminal semantic processing, which occurs in the near-absence of attention. The findings add solid evidence to the robustness and flexibility of unconscious processing and propose a revision of the Global Workspace Theory. Third, subliminal semantic processing, attentional status, and the influence of cue color can be revealed by cursor motion features, such as the curvature of trajectories, demonstrating that the mouse-tracking technique is a promising tool to reveal dynamic implicit cognition on a broad range of topics in future research.

## Figures and Tables

**Table 1:** Regressions with congruency effects ^1^ on *d*’s in the green condition.

	Predictor	*b*	*SE*	95% CI	*t*	*p*
Cued	(intercept)	1251.03	198.07	[862.81, 1639.25]	6.32 ***	<0.001
	*d*’	0.17	0.16	[−0.16, 0.50]	1.02	0.313
Uncued	(intercept)	479.97	133.92	[217.49, 742.45]	3.58 **	0.001
	*d*’	0.13	0.17	[−0.19, 0.46]	0.80	0.429

^1^ The congruency effect = AUC_incongruent_ − AUC_congruent_. ** *p* < 0.01, two-tailed. *** *p* < 0.001, two-tailed.

**Table 2:** Regressions with congruency effects on *d*’s in the red condition.

	Predictor	*b*	*SE*	95% CI	*t*	*p*
Cued	(intercept)	1014.28	297.93	[430.34, 1598.22]	3.40 **	0.002
	*d*’	−0.01	0.17	[−0.35, 0.34]	−0.03	0.973
Uncued	(intercept)	474.07	132.30	[214.76, 733.38]	3.57 **	0.001
	*d*’	0.04	0.16	[−0.28, 0.36]	0.25	0.807

** *p* < 0.01, two-tailed.

## Data Availability

The data presented in this study are available on request from the corresponding author.
